# Genetic Variants Involved in the Crystallization Pathway Are Associated with Calcium Nephrolithiasis in the Chinese Han Population

**DOI:** 10.3390/genes13060943

**Published:** 2022-05-25

**Authors:** Lujia Wang, Xiaoling Lin, Zijian Zhou, Yuanyuan Yang, Peng Gao, Zhong Wu

**Affiliations:** 1 Department of Urology, Huashan Hospital & Institute of Urology, Fudan University, Shanghai 200040, China; lukewang2006@126.com (L.W.); zjzhou21@m.fudan.edu.cn (Z.Z.); yyyang20@fudan.edu.cn (Y.Y.); gaopeng76@163.com (P.G.); 2 Clinical Research Center of Urolithiasis, Shanghai Medical College, Fudan University, Shanghai 200040, China; 3 Department of Urology, Shanghai Children’s Hospital, School of Medicine, Shanghai Jiao Tong University, Shanghai 200062, China; lxling.85@163.com

**Keywords:** nephrolithiasis, polymorphism, calcium metabolism, crystallization pathway, Chinese Han population

## Abstract

A genome-wide association analysis study (GWAS) in the Japanese population identified 14 significant loci associated with nephrolithiasis. Besides 4 novel loci related to metabolic traits, the 10 remaining loci were associated with kidney or electrolyte-related traits. We aimed to replicate the association of these loci with calcium nephrolithiasis in the Chinese Han population. A case–control association analysis was conducted involving 691 calcium nephrolithiasis patients and 1008 control subjects. We were able to genotype a total of 11 single-nucleotide polymorphisms (SNPs) previously identified as being correlated with nephrolithiasis in the Japanese population. SNP rs35747824 at *PDILT* was related to calcium nephrolithiasis in the Chinese Han population (*p* = 4.346 × 10^−3^, OR = 1.292). Moreover, four SNPs at four loci, rs6667242 at *ALPL* (*p* = 0.02999, OR = 0.8331), rs1544935 at *KCNK5* (*p* = 0.01341, OR = 0.7804), rs7328064 at *DGKH* (*p* = 0.007452, OR = 1.211) and rs13041834 at *BCAS1* (*p* = 0.03897, OR = 0.8409), were suggestively associated with calcium nephrolithiasis. Our results demonstrated that the genetic variants at 1p36.12, 6p21.2, 13q14.11, 16p12.3 and 20q13.2 are associated with calcium nephrolithiasis in the Chinese Han population. Furthermore, our study highlights the importance of genetic variance associated with the crystallization pathway in Chinese patients with calcium nephrolithiasis.

## 1. Introduction

Nephrolithiasis is a common disorder among all populations. It has been reported that the prevalence of nephrolithiasis is about 10–15% in males and 7% in females [1]. Most kidney stones are composed of calcium (70–85%), complexed to either oxalate or phosphate [2]. In urine, calcium renal calculus is formed when the relative concentrations of lithogenic substances and inhibitors of stone formation are imbalanced, which may result in crystal precipitation and aggregation [3]. It should be noted that in up to 50% of individuals, nephrolithiasis recurs within 10 years of the initial episode, and a decline in renal function has been associated with recurrent stone disease [4]. The etiology of nephrolithiasis and associated metabolic abnormalities is multifactorial, including diet, ethnicity, genetics and environmental factors [5]. Twin studies have reported heritabilities of >45% and >50% for nephrolithiasis and hypercalciuria, respectively [6,7]. Individuals with a strong family history of urolithiasis, including a parent and a sibling, have a standardized incidence ratio of >50% for developing stones [8], indicating the pivotal role of genetic factors.

Up until now, seven genome-wide association studies (GWASs) in European and Japanese populations have reported associations of nephrolithiasis with 25 loci [9,10,11,12,13,14]. In the GWAS conducted by Tanikawa et al. [14] in the Japanese population, they identified 14 significant loci, including 9 novel loci. Four novel loci were related to metabolic traits, whereas the remaining 10 loci were associated with kidney or electrolyte-related traits which may impact the crystallization pathways of urolithiasis. In this study, we conducted a replication study regarding the association between polymorphisms at these loci and the risk of calcium nephrolithiasis in the Chinese Han population. We believe that our results may help to elucidate the molecular pathology of calcium nephrolithiasis in Chinese patients.

## 2. Material and Methods

### 2.1. Ethics Statement

The protocol for this study was compliant with the Declaration of Helsinki. All participants gave their written informed consent before participating in this study. Approval of the research protocol was obtained from the Huashan Institutional Review Board of Fudan University (HIRB).

### 2.2. Subjects

In total, 691 unrelated Chinese Han patients with nephrolithiasis (467 males and 224 females, mean age: 50.47 years) were recruited at Huashan Hospital of Fudan University. The procedure eliminated patients with radioparent stones, such as cystine, struvite and uric acid stones. Moreover, secondary nephrolithiasis in patients with known causes, including gout, renal failure, hyperparathyroidism, osteoporosis or cancer were excluded. Also excluded were those taking calcium and/or vitamin D supplements or taking medications that may affect urinary calcium excretion. In the control group, there were 1,008 subjects without a family history of nephrolithiasis or stone disease. Calcium nephrolithiasis was diagnosed clinically either with plain kidney–ureter–bladder (KUB) radiography or a non-contrast computed tomography (NCCT) scan. All the patients with calcium nephrolithiasis and the control subjects belonged to the same racial, ethnic, geographical and environmental strata.

We assessed the effect of genetic variations on serum sodium, potassium, calcium, magnesium, phosphorus, chloride, carbon dioxide (CO_2_), creatinine, urea, uric acid, alkaline phosphatase (ALP), parathyroid hormone (PTH), albumin, glucose, 25(OH)D_3_, cholesterol, triglyceride, low-density lipoprotein (LDL), high-density lipoprotein (HDL), urine calcium and phosphorus levels, as well as estimated glomerular filtration rate (eGFR) and body mass index (BMI).

### 2.3. SNP Selection and Genotyping

A standard quality control procedure was applied to select SNPs for further analysis. SNPs with the following conditions were excluded: (i) genotype call rate < 90%; (ii) minor allele frequency (MAF) < 0.01; or (iii) *p* < 0.001 for the Hardy–Weinberg equilibrium (HWE) test. We were able to genotype a total of 17 single-nucleotide polymorphisms (SNPs) significantly associated with nephrolithiasis in GWAS.

### 2.4. Statistical Analysis

Quantitative data were expressed as mean ± standard deviation (SD). Continuous variables were tested with an independent *t*-test. The chi-square test was used to analyze categorical variables. Genotype distributions for the SNPs were tested for Hardy–Weinberg equilibrium (HWE). SNP associations with nephrolithiasis were examined by a Cochran–Armitage trend test. Results are expressed as odds ratios (ORs) and 95% confidence intervals (CI). A *p*-value lower than 4.545 × 10^−3^ (0.05/11) was considered statistically significant. SNPs with a *p*-value less than 0.05 were also considered of interest. Multiple logistic regression analyses were conducted to investigate the relationship between genotype and recurrent disease, hypertension and diabetes mellitus. Genotype and clinical parameters were tested through multiple linear regression analyses. We conducted association and QTL analyses using the PLINK-1.07 toolset. *p*-values were two-tailed. An α of 0.05 indicated statistical significance.

## 3. Results

Table 1 showed the clinical characteristics of the case and control samples. Compared with the healthy controls, body mass index (BMI) was significantly higher in patients with calcium nephrolithiasis (*p* = 0.003). No significant difference was observed in the distribution of serum calcium, phosphorus, magnesium, creatinine, uric acid, albumin, triglycerides and cholesterol between the patients and controls.

Table 2 shows the genotype frequencies of polymorphism among all subjects. The genotype frequencies of 11 SNPs were distributed in accordance with the Hardy–Weinberg equilibrium. The SNP rs35747824 at *PDILT* had a significant association with calcium nephrolithiasis in the Chinese Han population (*p* = 4.346 × 10^−3^, OR = 1.292). Moreover, four SNPs at four loci, rs6667242 at ALPL (*p* = 0.02999, OR = 0.8331), rs1544935 at KCNK5 (*p* = 0.01341, OR = 0.7804), rs7328064 at DGKH (*p* = 0.007452, OR = 1.211) and rs13041834 at BCAS1 (*p* = 0.03897, OR = 0.8409), were suggestively associated with the risk of calcium nephrolithiasis. The A allele of rs6667242, the T allele of rs1544935, the C allele of rs7328064, the T rs35747824 and the T allele of rs13041834 were found to increase the risk of nephrolithiasis development.

Next, we examined the association of these significant SNPs with several clinical parameters. As shown in Table 3, there were no significant SNPs related to recurrent nephrolithiasis disease, hypertension or diabetes mellitus. As shown in Table 4, the T allele of rs35747824 was significantly correlated with higher levels of serum urea (*p* = 0.01471) and urine phosphorus (*p* = 0.01966). The T allele of rs1544935 was significantly correlated with a lower level of urine calcium (*p* = 0.03293). The T allele of rs13041834 was significantly correlated with a higher level of serum magnesium (*p* = 0.008117).

## 4. Discussion

In 2019, Tanikawa et al. [14] conducted a GWAS for urolithiasis in the Japanese population which included more than 13,000 cases and 190,000 control samples. They found 14 significant loci, including 9 novel loci, that were associated with the risk of urolithiasis in the Japanese population. Four novel loci (2p23.3, 6p12.3, 16q12.2 and 17q23.2) could potentially promote the formation of urolithiasis through the regulation of metabolic traits, such as obesity and increased levels of uric acid. Four previously reported loci (5q35.3, 7q14.3 13q14.11 and 21q22.13) and three of the novel loci (6q23.2, 20q13.2 and 19q13.12) were associated with the regulation of serum and urine calcium levels. The other three loci (1p36.12, 6p21.2 and 16p12.3) were speculated to regulate the crystallization step of calcium nephrolithiasis. Thus, all 14 loci were speculated to be associated with the regulation of either the metabolic or crystallization pathways of lithogenesis. In this study, we evaluated the association of 11 single-nucleotide polymorphisms (SNPs) at 11 loci identified in former GWASs as being related to nephrolithiasis in the Chinese Han population. We demonstrated that rs6667242 at 1p36.12, rs1544935 at 6p21.2, rs7328064 at 13q14.11, rs35747824 at 16p12.3 and rs13041834 at 20q13.2 were associated with risk of calcium nephrolithiasis in Chinese patients.

rs35747824 is located in the intron of *PDILT*, within the *UMOD*-*PDILT* locus at 16p12.3. *UMOD* encodes uromodulin, the most abundant protein in urine and a known inhibitor of calcium–phosphate precipitation. Studies have shown that *UMOD* knockout mice had lower creatinine clearance and were more susceptible to urinary tract infections [15,16]. In a meta-analysis study of common variants in *UMOD*, the adjacent gene *PDILT* (protein disulfide isomerase-like, testis expressed) was significantly associated with urinary uromodulin levels [17]. Another meta-analysis indicated that SNPs within *PDILT*-*UMOD* were associated with rapid kidney function decline [18]. Furthermore, a common variant at *UMOD* has been identified as a protective SNP against nephrolithiasis in a GWAS [10]. In the GWAS by Tanikawa et al. [14], rs35747824 was significantly associated with urolithiasis in the Japanese population. The authors suggested that rs35747824 was associated with kidney-related traits, since the allele of rs35747824 was correlated with higher serum urea, creatinine and uric acid and lower eGFR. In our study, rs35747824 was significantly associated with risk of calcium nephrolithiasis in the Chinese Han population. The risk allele rs35747824 was correlated with higher levels of serum urea and urine phosphorus, which suggested that the *PDILT*-*UMOD* locus was not only associated with kidney-related traits but also with electrolyte-related traits. It has been proven that high urine phosphate is an important risk factor for calcium nephrolithiasis. It was reported that increased phosphaturia could upregulate 1-α hydroxylase activity and that it increased 1,25 vitamin D, which ultimately led to hypercalciuria and calcium nephrolithiasis [19].

rs1544935 at 6p21.2 is located within *KCNK5*, which encodes potassium two pore domain channel subfamily K member 5 (KCNK5). KCNK5 is strongly expressed in renal proximal tubules and papillary collecting ducts. *KCNK5* knockout mice exhibit metabolic acidosis due to renal loss of carbonate [20]. In the kidneys, *KCNK5* plays an important role in maintaining normal levels of plasma potassium, as well as exerting various functions, including cell volume control, membrane potential stabilization and excitability, and regulation of hormone or ion secretion [21]. However, the role of the *KCNK5* gene polymorphism in calcium nephrolithiasis has not been fully elucidated. In a GWAS in British and Japanese populations by Howles et al. [13], rs1155347 at *KCNK5* was associated with nephrolithiasis in a trans-ethnic meta-analysis. In a GWAS in the Japanese population, rs1544935 at *KCNK5* was identified as being related to risk of urolithiasis [14] and the authors presumed that this variant might promote stone formation by regulating urine pH levels. In the present study, rs1544935 showed a suggestive significant association with calcium nephrolithiasis in the Chinese Han population.

rs13041834 at 20q13.2 is located near *CYP24A1*, which encodes cytochrome P450 family 24 subfamily A member 1 (CYP24A1), an enzyme that metabolizes active 1,25-dihydroxyvitamin D into inactive 24,25-dihydroxyvitamin D. It was reported that loss-of-function mutations in *CYP24A1* caused autosomal recessive infantile hypercalcemia type 1 (OMIM 126065) [22]. In the GWAS by Howles et al. [13], a genome-wide significant SNP associated with nephrolithiasis, rs17216707, was identified in British and Japanese populations; the allele is ~38 kb upstream of *CYP24A1*. Individuals homozygous for the *CYP24A1* risk allele rs17216707-T had significantly higher serum calcium concentrations than heterozygotes [13]. In the GWAS by Tanikawa et al. [14], among the Japanese population, rs13041834 obviously increased the risk of urinary calculi. In our study, rs13041834 showed a suggestive association with calcium nephrolithiasis in the Chinese Han population, and the risk allele rs13041834 was correlated with higher levels of serum magnesium. Since magnesium is well maintained in the body by the kidneys, we speculated that higher serum magnesium levels could be attributed to the impaired urine concentration affected by rs13041834. As an inhibitor of kidney stones, magnesium competes with calcium to bind oxalates in urine and the ratio of magnesium/calcium in urine is correlated with risk of nephrolithiasis [23].

*ALPL* encodes the alkaline phosphatase tissue-nonspecific isozyme (ALPL), which is expressed in the proximal tubules of the kidney [24]. In the GWAS by Oddsson et al. [12] in Icelanders, they identified protective and risk genes for nephrolithiasis that were correlated with elevated and reduced serum ALP levels, respectively. As alkaline phosphatase (ALP) hydrolyzes pyrophosphate into free phosphate, the risk of nephrolithiasis associated with the variants of *ALPL* should be dependent on the balance between stone-inhibiting pyrophosphate and phosphate in the kidneys [25]. Li et al. [26] replicated the GWAS in Icelanders and identified rs1256328 at *ALPL* as being associated with nephrolithiasis in the Chinese Han population. In the GWAS by Tanikawa et al. [14], rs6667242 at 1p36.12 showed a significant association with urolithiasis in the Japanese population. The risk allele rs6667242 was associated with higher serum ALP levels and lower serum phosphate levels. Although we observed the association of rs6667242 with calcium nephrolithiasis in the Chinese Han population in the present study, the risk allele rs6667242 was not correlated with any of the electrolyte traits.

*DGKH* encodes for diacylglycerol kinase eta (DGKH). In the GWAS by Urabe et al., they first identified *DGKH* at 13q14.11 as being associated with calcium nephrolithiasis in the Japanese population [11]. However, we failed to replicate the finding of an association of rs7981733, rs4142110 and rs17646069 with *DGKH* in the Chinese Han population in our previous study [27]. In the GWAS of Howles et al. [13], an intronic variant in *DGKH*, rs1037271, was associated with nephrolithiasis in British and Japanese populations, and the authors suggested that SNPs of *DGKH* were predicted to promote kidney stone formation by influencing CaSR signaling. In the GWAS by Tanikawa et al. [14], it was reported that rs7328064 in *DGKH* was significantly associated with urolithiasis in the Japanese population. The risk allele rs7328064 was correlated with lower levels of serum creatinine and higher eGFRs, which demonstrated that rs7328064 might also be associated with kidney-related traits. In our results, rs7328064 showed a suggestive association with calcium nephrolithiasis in the Chinese Han population.

**Figure 1:**
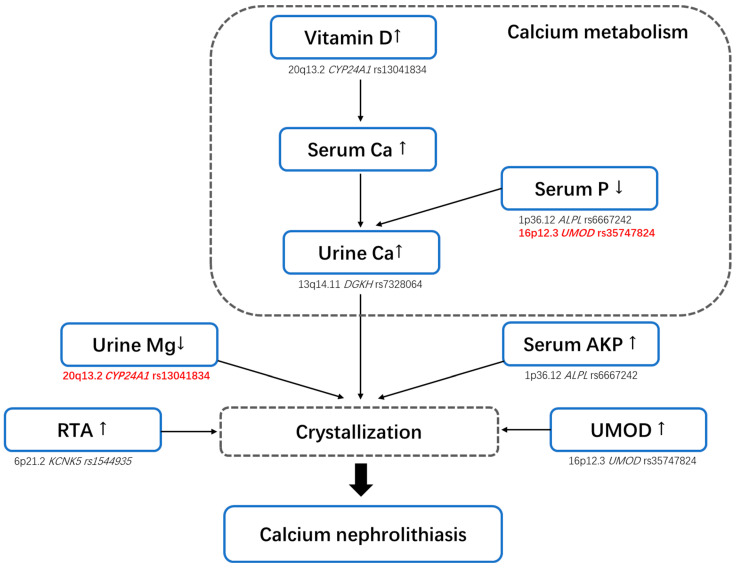
Schematic of the related components of the kidney calcium metabolism and crystallization pathways considered in the present study, including the putative effector genes and corresponding SNPs. The newly discovered correlations are shown in red.

In the present study, we were able to replicate 11 SNPs discovered in the GWAS of nephrolithiasis by Tanikawa et al. [14]. To our knowledge, there are no similar studies replicating the results of their GWAS in the Chinese Han population. It is worth noting that most of the significant SNPs validated in our study were involved in calcium metabolism or crystallization in the kidneys, rather than the metabolic pathway (Figure 1). This discrepancy can be partly explained by the fact that stone composition in our study was limited to calcium-containing stones. Remarkably, our results indicated the important role of genetic variance associated with calcium metabolism and the crystallization pathway in calcium nephrolithiasis.

## 5. Conclusions

To conclude, our results demonstrated that genetic variants at 1p36.12, 6p21.2, 13q14.11, 16p12.3 and 20q13.2 are associated with calcium nephrolithiasis in the Chinese Han population. Since the variants at 13q14.11 and 20q13.2 are related to calcium metabolism in the kidneys and the variants at 1p36.12, 6p21.2 and 16p12.3 are speculated to regulate the crystallization step of lithogenesis, our results highlight the importance of genetic variance associated with the crystallization pathway in Chinese patients with calcium nephrolithiasis.

## Figures and Tables

**Table 1:** Baseline characteristics of the study population. The bold means statistically significant difference.

Parameters	Cases (*n* = 691)	Controls (*n* = 1008)	*p*-Value
Gender (male/female)	467/224	705/303	0.302
Age (years)	50.47 ± 12.55	49.98 ± 12.65	0.431
BMI (kg/m^2^)	24.76 ± 3.30	24.28 ± 3.26	**0.003**
Stone frequency (primary/recurrence)	418/273	-	-
Serum calcium (mmol/L)	2.26 ± 0.12	2.27 ± 0.10	0.062
Serum phosphorus (mmol/L)	1.13 ± 0.21	1.14 ± 0.18	0.293
Serum magnesium (mmol/L)	0.91 ± 0.93	0.89 ± 0.75	0.625
Serum creatinine (μmol/L)	85.19 ± 41.93	83.64 ± 23.51	0.331
Serum uric acid (mmol/L)	0.360 ± 0.088	0.353 ± 0.077	0.083
Serum albumin (g/L)	42.11 ± 4.11	41.86 ± 4.09	0.217
Serum triglycerides (mmol/L)	1.59 ± 0.80	1.62 ± 0.84	0.461
Serum cholesterol (mmol/L)	4.60 ± 1.24	4.65 ± 1.22	0.410

**Table 2:** Results of the association analysis for calcium nephrolithiasis in the Chinese Han population.

Chr.	SNP	Region	Gene	Alleles		Cases				Controls				*p*	OR	95%CI
				Minor	Major	n(11) ^a^	n(12)	n(22)	MAF	n(11)	n(12)	n(22)	MAF			
1	rs6667242	1p36.12	*ALPL*	G	A	34	219	297	0.2609	83	434	491	0.2976	**0.02999**	0.8331	0.7064–0.9826
2	rs1260326	2p23.3	*GCKR*	C	T	142	328	221	0.4428	207	507	293	0.4573	0.4054	0.9432	0.8219–1.082
5	rs11746443	5q35.3	*RGS14*	A	G	21	222	448	0.191	39	271	698	0.1731	0.1822	1.128	0.945–1.346
6	rs1544935	6p21.2	*KCNK5*	G	T	12	154	498	0.134	26	281	699	0.1655	**0.01341**	0.7804	0.641–0.9502
6	rs3798519	6p12.3	*TFAP2B*	C	A	48	244	371	0.2564	46	358	566	0.232	0.109	1.142	0.9708–1.343
6	rs6928986	6q23.2	*EPB41L2*	C	T	109	296	238	0.3997	152	483	354	0.3979	0.9177	1.008	0.873–1.163
7	rs6975977	7p14.3	*INMT-FAM188B*	A	G	7	105	553	0.08947	15	180	797	0.1058	0.1223	0.8301	0.6553–1.052
13	rs7328064	13q14.11	*DGKH*	C	A	171	347	160	0.5081	179	487	252	0.4602	**0.007452**	1.211	1.053–1.394
16	rs35747824	16p12.3	*PDILT*	T	A	32	222	430	0.2091	37	252	671	0.1698	0.004346 *	1.292	1.083–1.542
19	rs74956940	19p13.12	*PKN1*	G	C	25	183	395	0.1932	32	298	624	0.1897	0.8102	1.023	0.8516–1.228
20	rs13041834	20q13.2	*BCAS1*	C	T	26	241	415	0.2148	62	365	569	0.2455	**0.03897**	0.8409	0.7132–0.9914

Abbreviations: Chr., chromosome; CI, confident interval; MAF, minor allele frequency; OR, odds ratio; SNP, single-nucleotide polymorphism. ^a^ n(11), number of subjects with homozygous genotypes for the minor allele; n(12), number of subjects with heterozygous genotypes; n(22), number of subjects with homozygous genotypes for the major allele. * *p* < 0.05/11

**Table 3:** Multiple logistic regression analyses for clinical parameters.

SNP	Recurrent Disease			Hypertension			Diabetes Mellitus		
	OR	s.e. ^b^	*p*	OR	s.e.	*p*	OR	s.e.	*p*
rs6667242	0.8	0.2441	0.3601	1.227	0.2345	0.3824	1.075	0.3571	0.8405
rs1544935	1.411	0.2953	0.2417	0.8592	0.3074	0.6211	1.352	0.4152	0.4664
rs7328064	0.9796	0.1994	0.9177	0.7451	0.1992	0.1393	1.176	0.3007	0.5894
rs35747824	1.072	0.2453	0.7766	0.9031	0.2476	0.6807	0.7811	0.3871	0.5224
rs13041834	0.7783	0.2465	0.3084	1.113	0.2373	0.6518	0.9484	0.3609	0.8832

Abbreviations: SNP, single-nucleotide polymorphism; OR, odds ratio. ^b^ s.e., standard error of mean.

**Table 4:** Multiple linear regression analyses for clinical parameters.

	rs6667242			rs1544935			rs7328064			rs35747824			rs13041834		
	Beta ^c^	s.e. ^d^	*p*	Beta	s.e.	*p*	Beta	s.e.	*p*	Beta	s.e.	*p*	Beta	s.e.	*p*
eGFR ^e^	1.594	3.844	0.6796	2.419	5.014	0.6308	−1.834	3.381	0.5891	2.161	3.823	0.5734	−0.654	3.587	0.8558
Serum creatinine	1.661	4.136	0.6884	−4.099	5.342	0.4438	4.984	3.665	0.1753	−0.9605	4.39	0.827	−4.37	4.222	0.3018
Serum urea	−0.3615	0.5457	0.5085	−0.02013	0.3084	0.948	−0.2489	0.4711	0.5978	1.365	0.5549	**0.01471**	−0.765	0.5394	0.1575
Serum uric acid	0.006173	0.01069	0.5643	−0.01249	0.01372	0.3636	0.006789	0.009428	0.4723	0.02088	0.0112	0.06371	0.01506	0.01083	0.1656
Serum sodium	0.1369	0.2913	0.6389	−0.1459	0.3693	0.6933	−0.3738	0.252	0.1394	0.1134	0.3035	0.709	−0.06286	0.2926	0.8301
Serum potassium	0.2138	0.3062	0.4858	−0.1813	0.3805	0.6342	0.43	0.2603	0.09995	−0.2452	0.312	0.4327	0.234	0.3006	0.4371
Serum calcium	−0.00702	0.01335	0.5996	−0.01898	0.01705	0.2667	0.01394	0.01172	0.2355	0.01066	0.01401	0.4473	−0.009201	0.0135	0.4964
Serum magnesium	−0.17	0.1978	0.3912	0.3997	0.2447	0.1039	0.007906	0.1692	0.9628	−0.1604	0.2013	0.4267	0.51	0.1908	**0.008117**
Serum phosphorus	−0.02663	0.0203	0.1912	0.02217	0.02614	0.3974	−0.004899	0.018	0.7858	0.01873	0.02136	0.3816	0.02346	0.02064	0.2571
Serum chloride	1.586	1.351	0.2431	1.102	1.826	0.5474	0.3793	1.205	0.7535	0.4335	1.586	0.7851	1.012	1.515	0.5055
Serum CO_2_	−0.4642	0.3749	0.2171	0.6859	0.4757	0.1507	−0.08025	0.3284	0.8072	0.1175	0.3898	0.7633	0.7341	0.3747	0.05135
Serum ALP	−3.448	3.492	0.3247	−7.364	4.454	0.0997	4.283	3.069	0.1643	−1.573	3.678	0.6693	−6.496	3.517	0.06609
PTH	10.5	15.4	0.5015	−3.914	21.34	0.8558	−6.033	14.16	0.6733	0.01759	15.14	0.9991	−15.24	15.92	0.3467
Serum 25(OH)D3	−0.8105	5.833	0.8906	9.349	8.259	0.2676	−0.7929	4.926	0.8733	−3.922	6.088	0.5249	5.433	6.045	0.3767
BMI	−0.4291	0.3849	0.2662	−0.4376	0.5037	0.386	−0.2049	0.3431	0.5511	0.1449	0.4138	0.7266	−0.2607	0.3986	0.5137
Serum albumin	−0.2966	0.4241	0.4852	−0.2503	0.546	0.6471	0.1871	0.377	0.6203	0.2914	0.4495	0.5175	−0.06749	0.4336	0.8765
Serum glucose	−0.2328	0.1266	0.06755	0.08015	0.2012	0.6908	−0.0169	0.1355	0.9009	−0.1584	0.16	0.3232	−0.05665	0.1545	0.7142
Serum total cholesterol	−0.364	0.783	0.6492	1.083	0.6451	0.1139	0.4715	0.6727	0.4942	−0.9325	0.8684	0.3011	−1.063	0.8388	0.2242
Serum triglycerides	0.014	0.2162	0.9493	0.1763	0.1836	0.3521	−0.04755	0.1835	0.7991	−0.2038	0.2344	0.3992	−0.04	0.2372	0.8684
Serum LDL	−0.5435	0.6112	0.3889	0.6954	0.5457	0.2219	0.2475	0.5549	0.6619	−0.8462	0.7018	0.2479	−0.9212	0.6807	0.196
Serum HDL	0.1274	0.09721	0.2126	0.07761	0.09409	0.4233	0.07893	0.08858	0.388	0.02964	0.1238	0.8145	−0.04367	0.1187	0.7185
Urine calcium	0.15	0.9464	0.8765	−3.358	1.419	**0.03293**	0.3711	0.9964	0.7151	−0.9279	1.216	0.4591	−0.8074	1.174	0.5029
Urine phosphorus	0.6714	4.999	0.8952	−11.72	5.798	0.06287	4.387	3.75	0.2617	10.46	3.934	**0.01966**	1.947	4.655	0.6821
Urine pH	−0.04856	0.08224	0.5555	0.005251	0.1041	0.9598	−0.0004832	0.07171	0.9946	−0.005472	0.08558	0.9491	−0.02474	0.08241	0.7643

Abbreviations: eGFR, estimated glomerular filtration rate; ALP, alkaline phosphatase; PTH, parathyroid hormone; 25(OH)D3, 25-hydroxycholecalciferol; BMI, body mass index; LDL, low-density lipoprotein; HDL, high-density lipoprotein. ^c^ Beta, Regression coefficient. ^d^ s.e., standard error of mean. ^e^ eGFR (mL/min/1.73 m^2^) = 186 × (serum creatinine/88.41)^−1.154^ × age^−0.203^ (×0.742 if female). The bold ***p*** means statistically significant difference.

## Data Availability

Data could be made available upon reasonable request to the authors.
